# Finite-time analysis of epidemic reaction-diffusion models: Stability, synchronization, and numerical insights

**DOI:** 10.1371/journal.pone.0321132

**Published:** 2025-05-27

**Authors:** Iqbal Batiha, Nidal Anakira, Issam Bendib, Adel Ouannas, Amel Hioual, Irianto Irianto, Ala Amourah

**Affiliations:** 1 Department of Mathematics, Al Zaytoonah University of Jordan, Amman, Jordan; 2 Nonlinear Dynamics Research Center (NDRC), Ajman University, Ajman, United Arab Emirates; 3 Faculty of Education and Arts, Sohar University, Sohar, Oman; 4 Jadara Research Center, Jadara University, Irbid, Jordan; 5 Department Applied Mathematics, Brothers Mentouri University of Constantine, Constantine, Algeria; 6 Department of Mathematics and Computer Science, University of Oum EL-Bouaghi, Oum EL-Bouaghi, Algeria; 7 Department General Education, Faculty of Resilience, Rabdan Academy, Abu Dhabi, United Arab Emirates; 8 Applied Science Research Center, Applied Science Private University, Amman, Jordan; Lanzhou University of Technology, CHINA

## Abstract

This study presents an innovative approach to analyzing finite-time stability (FTS) and synchronization (FTSYN) in integer-order reaction-diffusion systems (RDs), particularly in the context of epidemiological modeling. By integrating Gronwall’s inequality, Lyapunov functionals (LFs), and linear control strategies, a comprehensive framework is developed to address transient dynamics within finite time frames. The proposed methodology advances the theoretical understanding of FTS and FTSYN by addressing the relatively unexplored dynamics of spatially extended systems. MATLAB simulations validate the theoretical findings, demonstrating the effectiveness of the control schemes and their practical applicability in modeling real-world disease transmission. Integrating spatial diffusion and disease dynamics provides critical insights into the influence of parameters such as diffusion rates and mortality on system behavior. This work contributes a robust framework for enhancing the analysis and management of nonlinear systems, with significant implications for epidemiology and other fields requiring rapid convergence and synchronization.

## Introduction

The study of infectious diseases has garnered considerable attention, with significant efforts directed toward advancing their understanding and management. This interest arises from the need to predict and mitigate disease transmission patterns to reduce mortality rates. Recent progress in mathematical modeling, particularly using the susceptible-infected-susceptible (SIS) epidemic RDs, demonstrates the potential for substantial reductions in disease-related fatalities [1–4]. De Jong *et al*. [5] introduced the standard incidence term βSI/N, diverging from the mass action principle and inspiring further investigations by Allen *et al*. into its broader applicability. Another study [6] proposed a frequency-dependent SIS RDs for continuous spatial domains, offering a refined approach to disease transmission by integrating spatial dynamics and interaction frequency. Peng and Liu [7] rigorously analyzed the endemic equilibrium in Allen *et al*.’s framework, elucidating its stability conditions and advancing the understanding of disease persistence and transmission. These findings form a robust foundation for designing effective intervention strategies, with implications for improving public health outcomes.

This study analyzes a RDs (1) described by:

$$\left\{ \begin{array}{cc} \frac{\partial S_{1}\left(x,t\right)}{\partial t}=d_{1}\Delta S_{1}+\Lambda -\beta \frac{S_{1}\varphi \left(I_{1}\right)}{S_{1}+I_{1}}-\mu S_{1}, & x\in \Omega ,t>0,\\ \frac{\partial I_{1}\left(x,t\right)}{\partial t}=d_{2}\Delta I_{1}+\beta \frac{S_{1}\varphi \left(I_{1}\right)}{S_{1}+I_{1}}-\left(\mu +\sigma \right)I_{1}, & x\in \Omega ,t>0,\\ \frac{\partial S_{1}}{\partial \tau }=\frac{\partial I_{1}}{\partial \tau }=0, & x\in \partial \Omega ,\\ S_{1}\left(x,0\right)=S_{1,0}\left(x\right)>0,\;I_{1}\left(x,0\right)=I_{1,0}\left(x\right)>0, & x\in \Omega . \end{array}\right.$$ (1)

The function $\varphi \left(I_{1}\right)$ is a continuously differentiable function that is positive on the interval ${\mathbb{R}}_{+}^{*}$, satisfying:

$$\varphi \left(0\right)=0,\;\text{and}\;0<I_{1}\varphi^{\prime }\left(I_{1}\right)\leq \varphi \left(I_{1}\right)\;\text{for all}\;I_{1}>0.$$ (2)

**Fig 1:**
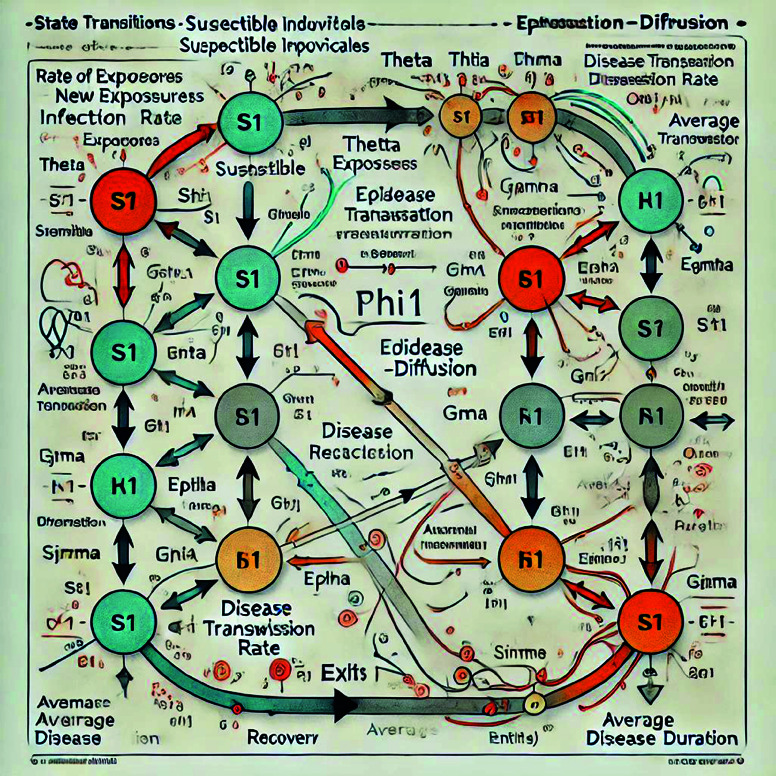
Flowchart illustrating transitions between ${𝚂}_{1}$ and ${𝙸}_{1}$ with parameter dependencies.

The terms and variables in the studied models significantly influence the system’s dynamics, as discussed in [8,9]. Key parameters include the following:

- The diffusion coefficients *d*_1_ and *d*_2_ represent the diffusion rates of susceptible and infected individuals across a spatial domain. These coefficients are influenced by real-world factors such as population mobility, migration, and travel patterns. Specifically, *d*_1_ denotes the rate at which susceptible individuals (e.g., uninfected people) move or are exposed to different areas, while *d*_2_ captures the rate at which infected individuals spread the disease spatially. For instance, *d*_2_ may be affected by the mobility of sick individuals traveling to hospitals or areas of high foot traffic, where disease transmission can occur more rapidly.
- The rate of new exposures (Λ) represents the frequency at which susceptible individuals come into contact with sources of infection, becoming exposed. This rate is influenced by social behavior, population density, and the effectiveness of public health interventions. For example, during a flu outbreak, Λ might be particularly high in crowded areas such as public transportation or schools, where encounters with infected individuals are more frequent.
- The disease spread frequency (β) governs the speed at which the infection spreads between individuals, making it a critical factor in understanding the growth of an epidemic. A higher β indicates faster disease transmission. This parameter is closely related to the transmission rate, which depends on factors such as the contagion level of the virus, its mode of transmission (e.g., airborne or surface contact), and protective measures like masks. Public health interventions, such as quarantine measures, vaccination campaigns, or isolation, can reduce β by limiting contact between susceptible and infected individuals.
- The mortality rate (μ) quantifies the rate at which infected individuals succumb to the disease. This rate reflects the disease’s lethality, depending on regional, population-specific, or healthcare-related contexts. For example, a more developed healthcare system may exhibit lower mortality rates due to better access to treatment. Additionally, factors such as the availability of medical resources, the severity of the disease, and population demographics (e.g., the higher vulnerability of older individuals) can significantly impact μ.
- The average disease duration (σ) describes the typical length of time an individual remains infectious and symptomatic. This duration is influenced by the progression of the disease and the availability of medical treatments. For example, individuals infected with influenza may be infectious for 5–7 days, whereas diseases like Ebola often involve longer contagious periods. Advances in medical treatments can shorten σ, while chronic illnesses or limited access to healthcare can prolong it, see Fig 1.

The study of stability theory remains a cornerstone in system analysis, with FTS gaining prominence due to its rapid convergence and enhanced robustness [10,11]. In [12], FTS for linear time-invariant fractional-order systems was introduced using the Lyapunov–Razumikhin technique [13]. In contrast, a novel criterion for FTS in integer-order nonlinear systems was proposed based on the generalized Gronwall inequality [14]. However, the absence of FTS equilibrium points (EPs) in most nonlinear systems underscores the inherent challenges in their stability analysis, emphasizing the need for innovative analytical techniques [15–23]. Synchronization of nonlinear systems has garnered significant attention over recent decades [24–31], with FTSYN emerging as a compelling research area [32]. Despite these advancements, the FTSYN of spatially extended systems, particularly RDs, remains under explored. This gap highlights an important avenue for future investigation into the dynamics and synchronization of such systems, with the potential to advance nonlinear dynamics and synchronization theory [33,34]. Recent developments underscore the role of finite-time control in managing nonlinear systems influenced by complex dynamics, such as event-triggered outputs [35]. This approach ensures that system states achieve desired behaviors or synchronization within a prescribed time, making it critical for applications requiring precision and rapid responses. Integer and fractional-order systems have also gained attention for their ability to model memory and hereditary effects prevalent in RD processes [36,37]. Event-triggered control reduces communication and computation overheads by activating control actions based on specific conditions, proving vital for large-scale networks like those in epidemiology and biology. Synchronization across integer and fractional-order RD nodes is crucial for maintaining coherence and stability in such systems [38,39]. Although advancements in synchronization techniques for nonlinear systems are notable, the FTSYN of integer-order RD networks remains relatively underexplored. This study addresses this gap by integrating finite-time control with integer-order dynamics to achieve synchronization in complex RD systems. The proposed approach provides valuable insights into the interplay between dynamics, synchronization, and FTS, with applications spanning epidemiology, physics, and engineering.

The paper is structured as follows: Mathematical background discusses the foundational mathematical framework and key theoretical tools required for the study. Finite time stability result focuses on analyzing the stability of EPs within a finite period, utilizing LF and related techniques. Finite-time synchronization scheme explores the synchronization dynamics of master-slave systems, emphasizing FTSYN with control strategies. Numerical simulations presents practical examples to validate theoretical results, showcasing the applicability of the proposed methods in real-world scenarios using MATLAB.

## Mathematical background

This section establishes the foundational mathematical framework and theoretical tools for analyzing FTS and FTSYN in RDss. Key concepts, such as LF, eigenvalue properties, and Gronwall’s inequality, are introduced to derivate stability conditions and synchronization criteria. Additionally, lemmas and definitions pertinent to the boundedness and convergence of solutions are presented to build a comprehensive theoretical basis for the subsequent sections.

**Lemma 1.** [40,41] *Assume that function S(t) satisfies:*

$$S\left(t\right)\leq p\left(t\right)+\int_{0}^{t}S\left(s\right)q\left(s\right)ds,\;t\in \left[0,t^{*}\right],$$ (3)

where $S\left(t\right),p\left(t\right),q\left(t\right)\in C\left[0,t^{*}\right],$ and p(t)≥0. If q(t) is non-decreasing, then

$$S\left(t\right)<p\left(t\right)e^{\int_{0}^{t}q\left(s\right)ds}.$$ (4)

**Lemma 2.** [42] *Let $𝚂(x)\in H_{0}^{1}(\Omega )$ satisfying the boundary condition $\frac{\partial 𝚂(x)}{\partial \eta }{|}_{\partial \Omega }=0$. Then, for the eigenvalue $\nu_{1}>0$ associated with the following system:*

$$\left\{ \begin{array}{cc} \nu_{1}S\left(x\right)=-\Delta S\left(x\right), & x\in \Omega ,\\ \frac{\partial S\left(x\right)}{\partial \eta }=0, & x\in \partial \Omega . \end{array}\right.$$ (5)

the following inequality holds:

$$\int_{\Omega }{\left|\nabla S\left(x\right)\right|}^{2}dx\geq \nu_{1}\int_{\Omega }{\left|S\left(x\right)\right|}^{2}\;dx.$$ (6)

**Lemma 3.** *If the subsequent conditions hold:*

- *There exist constant C_1_>0 such that the function φ satisfies the Lipschitz condition:*
  $$\left|\varphi \left(I_{1}\right)-\varphi \left(I_{2}\right)\right|\leq C_{1}\left|I_{1}-I_{2}\right|.$$ (7)
- *There exist constant C_2_>0 such that the function φ is uniformly bounded:*
  $$\left|\varphi \left(I_{1}\right)\right|\leq C_{2}.$$ (8)

*Then, the inequality (9) holds:*

$$\left|\frac{S_{1}\varphi \left(I_{1}\right)}{S_{1}+I_{1}}-\frac{S_{2}\varphi \left(I_{2}\right)}{S_{2}+I_{2}}\right|\leq C\left(\left|S_{1}-S_{2}\right|+\left|I_{1}-I_{2}\right|\right),$$ (9)

*where $C=C_{1}+C_{2}.$*

**Proof 1.** We estimate the term $\left|\frac{S_{1}\varphi \left(I_{1}\right)}{S_{1}+I_{1}}-\frac{S_{1}^{*}\varphi \left(I_{1}^{*}\right)}{S_{1}^{*}+I_{1}^{*}}\right|$ as follows:

$$\left|\frac{S_{1}\varphi \left(I_{1}\right)}{S_{1}+I_{1}}-\frac{S_{2}\varphi \left(I_{2}\right)}{S_{2}+I_{2}}\right|\leq \frac{\left|S_{1}\right|}{\left|S_{1}+I_{1}\right|}\left|\varphi \left(I_{1}\right)-\varphi \left(I_{2}\right)\right|+\left|\varphi \left(I_{2}\right)\right|\left|\frac{S_{1}}{S_{1}+I_{1}}-\frac{S_{2}}{S_{2}+I_{2}}\right|\;\leq \left|\varphi \left(I_{1}\right)-\varphi \left(I_{2}\right)\right|+\left|\varphi \left(I_{2}\right)\right|\frac{\left|S_{1}I_{2}-S_{2}I_{1}\right|}{\left|S_{1}+I_{1}\right|\left|S_{2}+I_{2}\right|}\;\leq C_{1}\left|I_{1}-I_{2}\right|+C_{2}\frac{\left|I_{2}\right|\left|S_{1}-S_{2}\right|+\left|S_{2}\right|\left|I_{1}-I_{2}\right|}{\left|S_{1}+I_{1}\right|\left|S_{2}+I_{2}\right|}\;\leq \left(C_{1}+C_{2}\right)\left(\left|S_{1}-S_{2}\right|+\left|I_{1}-I_{2}\right|\right)\;=C\left(\left|S_{1}-S_{2}\right|+\left|I_{1}-I_{2}\right|\right).$$ (10)

**Definition 1.** [40] *The system given by (1) is FTS with respect to {δ,ε,J},δ<ε, if ‖L(0)‖<δ implies ‖L(t)‖<ε,∀t∈J,*

*where $\left\|𝙻\left(0\right)\right\|=\max_{x\in \left[0,\ell \right]}\left|𝙻\left(0\right)\right|$, and $\left\|𝙻\left(t\right)\right\|=\max_{x\in \left[0,\ell \right]}\left|𝙻\left(\left(x,t\right)\right)\right|$.*

**Lemma 4.** [43] *The conditions stated in (2) imply*

$$0<\frac{\varphi \left(I_{1}\right)}{I_{1}}\leq \varphi^{\prime }\left(0\right),\;\text{for all}\;I_{1}>0.$$ (11)

## Finite time stability result

In this section, we analyze the FTS of the EPs for the proposed RD epidemic model. FTS ensures that system trajectories converge to the equilibrium states within a bounded time, which is critical for applications requiring rapid stabilization of dynamic processes.

Two key EPs are considered:

- The disease-free EP ${ℰ}_{0}^{*}=\left(\frac{\Lambda }{\mu },0\right),$ representing a scenario where the infection is eradicated from the population.
- The endemic EP ${ℰ}_{1}^{*}=\left(\frac{\Lambda -\left(\mu +\sigma \right){𝚅}_{1}^{*}}{\mu },{𝚅}_{1}^{*}\right),$ which characterizes the persistence of the infection in the population under certain conditions.

We employ LF, Gronwall’s inequality, and eigenvalue analysis to derive sufficient conditions for FTS at these EPs. The analysis focuses on the role of model parameters, such as diffusion rates, mortality, and recovery rates, in determining the EPs between the absence of the disease and its persistent presence in the population. Furthermore, explicit formulas for the settling time are presented, offering insights into the temporal dynamics of the system under different parameter settings.

**Theorem 1.** *The EP ${ℰ}_{0}$ of the system (1) is FTS, if the following conditions are satisfied:*

max{Ξ}>0, (12)

where $\Xi =\beta \varphi^{\prime }\left(0\right)-\nu_{1}\nu_{2}\left(q_{1}+q_{2}\right)-2\mu -\sigma ,\frac{\mu +\sigma }{2\mu }\left(\beta \varphi^{\prime }\left(0\right)\left(\frac{\left(d_{1}+d2\right)2}{2d_{1}d_{2}}+\frac{2\mu }{\mu +\sigma }\right)-\mu \right).$ Additionally, $t_{1}^{*}$ is defined as:

$${t_{1}}^{*}=\frac{1}{\max\left\{\Xi \right\}}\ln\left(\frac{\varepsilon }{\delta }\right).$$ (13)

where $\nu_{1}$ and $\nu_{2}$ are positive eigenvalues.

**Proof 2.** We utilize a positive LF defined as:

$$L_{1}\left(t\right)=\int_{\Omega }S_{1}I_{1}+\frac{{\left(d_{1}+d_{2}\right)}^{2}}{4d_{1}d_{2}}{\left(S_{1}-\frac{\Lambda }{\mu }\right)}^{2}+\frac{1}{2}I_{1}^{2}+\frac{2\Lambda }{\mu +\sigma }I_{1}\;dx.$$ (14)

Then,

$$\frac{\partial L_{1}\left(t\right)}{\partial t}=\int_{\Omega }S_{1}I_{1}+\frac{{\left(d_{1}+d_{2}\right)}^{2}}{4d_{1}d_{2}}{\left(S_{1}-\frac{\Lambda }{\mu }\right)}^{2}+\frac{1}{2}I_{1}^{2}+\frac{2\Lambda }{\mu +\sigma }I_{1}\;dx\;=\int_{\Omega }\left[I_{1}+\frac{{\left(d_{1}+d_{2}\right)}^{2}}{2d_{1}d_{2}}\left(S_{1}-\frac{\Lambda }{\mu }\right)\right]\frac{\partial S_{1}\left(x,t\right)}{\partial t}\;dx\;\;+\int_{\Omega }\left[S_{1}+I_{1}+\frac{2\Lambda }{\mu +\sigma }\right]\frac{\partial I_{1}\left(x,t\right)}{\partial t}\;dx\;=\int_{\Omega }\left[I_{1}+\frac{{\left(d_{1}+d_{2}\right)}^{2}}{2d_{1}d_{2}}\left(S_{1}-\frac{\Lambda }{\mu }\right)\right]\left(d_{1}\Delta S_{1}+\Lambda -\beta \frac{S_{1}\varphi \left(I_{1}\right)}{S_{1}+I_{1}}-\mu S_{1}\right)\;dx\;\;+\int_{\Omega }\left[S_{1}+I_{1}+\frac{2\Lambda }{\mu +\sigma }\right]\left(d_{2}\Delta I_{1}+\beta \frac{S_{1}\varphi \left(I_{1}\right)}{S_{1}+I_{1}}-\left(\mu +\sigma \right)I_{1}\right)\;dx\;={ℒ}_{1}(t)+{ℒ}_{2}(t).$$ (15)

Next, we express the functions as:

$${ℒ}_{1}(t)={𝙼}_{1}(t)+{𝙼}_{2}(t),\;{ℒ}_{2}(t)={𝚆}_{1}(t)+{𝚆}_{2}(t),$$ (16)

where

$$\left\{ \begin{array}{c} M_{1}\left(t\right)=d_{1}\int_{\Omega }\left[I_{1}+\frac{{\left(d_{1}+d_{2}\right)}^{2}}{2d_{1}d_{2}}\left(S_{1}-\frac{\Lambda }{\mu }\right)\right]\Delta S_{1}\;dx\\ M_{2}\left(t\right)=\int_{\Omega }\left[I_{1}+\frac{{\left(d_{1}+d_{2}\right)}^{2}}{2d_{1}d_{2}}\left(S_{1}-\frac{\Lambda }{\mu }\right)\right]\left(\Lambda -\beta \frac{S_{1}\varphi \left(I_{1}\right)}{S_{1}+I_{1}}-\mu S_{1}\right)\;dx\\ W_{1}\left(t\right)=d_{2}\int_{\Omega }\left[S_{1}+I_{1}+\frac{2\Lambda }{\mu +\sigma }\right]\Delta I_{1}\;dx\\ W_{2}\left(t\right)=\int_{\Omega }\left[S_{1}+I_{1}+\frac{2\Lambda }{\mu +\sigma }\right]\left(\beta \frac{S_{1}\varphi \left(I_{1}\right)}{S_{1}+I_{1}}-\left(\mu +\sigma \right)I_{1}\right)\;dx. \end{array}\right.$$ (17)

By applying Green’s formula, we simplify each term as follows:


$$M_{1}\left(t\right)=d_{1}\int_{\Omega }\left[{𝙸}_{1}+\frac{{\left(d_{1}+d_{2}\right)}^{2}}{2d_{1}d_{2}}\left({𝚂}_{1}-\frac{\Lambda }{\mu }\right)\right]\Delta {𝚂}_{1}\;dx$$

$$=-d_{1}\int_{\Omega }\nabla \left[{𝙸}_{1}+\frac{{\left(d_{1}+d_{2}\right)}^{2}}{2d_{1}d_{2}}\left({𝚂}_{1}-\frac{\Lambda }{\mu }\right)\right]\nabla {𝚂}_{1}\;dx$$

$$=-d_{1}\int_{\Omega }\nabla {𝙸}_{1}\nabla {𝚂}_{1}\;dx-\frac{{\left(d_{1}+d_{2}\right)}^{2}}{2d_{2}}\int_{\Omega }{\left(\nabla {𝚂}_{1}\right)}^{2}\;dx,$$

$$M_{2}\left(t\right)=\int_{\Omega }\left[{𝙸}_{1}+\frac{{\left(d_{1}+d_{2}\right)}^{2}}{2d_{1}d_{2}}\left({𝚂}_{1}-\frac{\Lambda }{\mu }\right)\right]\left(\Lambda -\beta \frac{{𝚂}_{1}\varphi \left({𝙸}_{1}\right)}{{𝚂}_{1}+{𝙸}_{1}}-\mu {𝚂}_{1}\right)\;dx$$

$$=-\mu \frac{{\left(d_{1}+d_{2}\right)}^{2}}{2d_{2}}\int_{\Omega }{\left({𝚂}_{1}-\frac{\Lambda }{\mu }\right)}^{2}\;dx\;\;-\mu \int_{\Omega }{𝚂}_{1}{𝙸}_{1}\;dx+\Lambda \int_{\Omega }{𝙸}_{1}\;dx-\beta \int_{\Omega }\frac{{𝚂}_{1}{𝙸}_{1}\varphi \left({𝙸}_{1}\right)}{{𝚂}_{1}+{𝙸}_{1}}\;dx\;\;-\beta \frac{{\left(d_{1}+d_{2}\right)}^{2}}{2d_{1}d_{2}}\int_{\Omega }\frac{{𝚂}_{1}^{2}\varphi \left({𝙸}_{1}\right)}{{𝚂}_{1}+{𝙸}_{1}}\;dx+\beta \frac{{\left(d_{1}+d_{2}\right)}^{2}}{2d_{1}d_{2}}\frac{\Lambda }{\mu }\int_{\Omega }\frac{{𝚂}_{1}\varphi \left({𝙸}_{1}\right)}{{𝚂}_{1}+{𝙸}_{1}}\;dx,$$

$$W_{1}\left(t\right)=d_{2}\int_{\Omega }\left[{𝚂}_{1}+{𝙸}_{1}+\frac{2\Lambda }{\mu +\sigma }\right]\Delta {𝙸}_{1}\;dx\;\;-d_{2}\int_{\Omega }\nabla \left[{𝚂}_{1}+{𝙸}_{1}+\frac{2\Lambda }{\mu +\sigma }\right]\nabla {𝙸}_{1}\;dx$$

$$=-d_{2}\int_{\Omega }\nabla {𝚂}_{1}\nabla {𝙸}_{1}\;dx-d_{2}\int_{\Omega }{\left|\nabla {𝙸}_{1}\right|}^{2}\;dx,$$

$$W_{2}\left(t\right)=\int_{\Omega }\left[{𝚂}_{1}+{𝙸}_{1}+\frac{2\Lambda }{\mu +\sigma }\right]\left(\beta \frac{{𝚂}_{1}\varphi \left({𝙸}_{1}\right)}{{𝚂}_{1}+{𝙸}_{1}}-\left(\mu +\sigma \right){𝙸}_{1}\right)\;dx$$

$$=-\left(\mu +\sigma \right)\int_{\Omega }{𝙸}_{1}^{2}\;dx-\left(\mu +\sigma \right)\int_{\Omega }{𝚂}_{1}{𝙸}_{1}\;dx-2\Lambda \int_{\Omega }{𝙸}_{1}\;dx\;\;+\beta \int_{\Omega }\frac{{𝚂}_{1}^{2}\varphi \left({𝙸}_{1}\right)}{{𝚂}_{1}+{𝙸}_{1}}\;dx+\beta \int_{\Omega }\frac{{𝚂}_{1}{𝙸}_{1}\varphi \left({𝙸}_{1}\right)}{{𝚂}_{1}+{𝙸}_{1}}\;dx+\frac{2\beta \Lambda }{\mu +\sigma }\int_{\Omega }\frac{{𝚂}_{1}\varphi \left({𝙸}_{1}\right)}{{𝚂}_{1}+{𝙸}_{1}}\;dx.$$

Combining the simplified expressions and applying Lemmas 1–4, we estimate:

$${𝙼}_{1}\left(t\right)+{𝚆}_{1}\left(t\right)=-\frac{{\left(d_{1}+d_{2}\right)}^{2}}{2d_{2}}\int_{\Omega }{\left|\nabla S_{1}\right|}^{2}\;dx-\left(d_{1}+d_{2}\right)\int_{\Omega }\nabla S_{1}\nabla I_{1}\;dx-d_{2}\int_{\Omega }{\left|\nabla I_{1}\right|}^{2}\;dx\;\leq -\frac{\nu_{1}{\left(d_{1}+d_{2}\right)}^{2}}{2d_{2}}\int_{\Omega }S_{1}^{2}\;dx-\nu_{1}\nu_{2}\left(d_{1}+d_{2}\right)\int_{\Omega }S_{1}I_{1}\;dx-\nu_{2}\int_{\Omega }I_{1}^{2}\;dx,\;\leq -\nu_{1}\nu_{2}\left(d_{1}+d_{2}\right)\int_{\Omega }S_{1}I_{1}\;dx-\nu_{2}\int_{\Omega }I_{1}^{2}\;dx\;{𝙼}_{2}\left(t\right)+{𝚆}_{2}\left(t\right)=-\mu \frac{{\left(d_{1}+d_{2}\right)}^{2}}{2d_{2}}\int_{\Omega }{\left(S_{1}-\frac{\Lambda }{\mu }\right)}^{2}\;dx-\left(\mu +\sigma \right)\int_{\Omega }I_{1}^{2}\;dx\;\;-\left(2\mu +\sigma \right)\int_{\Omega }S_{1}I_{1}\;dx-\Lambda \int_{\Omega }I_{1}\;dx-\beta \frac{d_{1}^{2}+d_{2}^{2}}{2d_{1}d_{2}}\int_{\Omega }\frac{S_{1}^{2}\varphi \left(I_{1}\right)}{S_{1}+I_{1}}\;dx\;\;+\frac{\beta \Lambda }{\mu }\left(\frac{{\left(d_{1}+d_{2}\right)}^{2}}{2d_{1}d_{2}}+\frac{2\mu }{\mu +\sigma }\right)\int_{\Omega }\frac{S_{1}\varphi \left(I_{1}\right)}{S_{1}+I_{1}}\;dx\;\leq -\mu \frac{{\left(d_{1}+d_{2}\right)}^{2}}{2d_{2}}\int_{\Omega }{\left(S_{1}-\frac{\Lambda }{\mu }\right)}^{2}\;dx-\left(\mu +\sigma \right)\int_{\Omega }I_{1}^{2}\;dx\;\;+\left(\beta \varphi^{\prime }\left(0\right)-2\mu -\sigma \right)\int_{\Omega }S_{1}I_{1}\;dx+\frac{\Lambda }{\mu }\left(\beta \varphi^{\prime }\left(0\right)\left(\frac{{\left(d_{1}+d_{2}\right)}^{2}}{2d_{1}d_{2}}+\frac{2\mu }{\mu +\sigma }\right)-\mu \right)\;\;\times \;\int_{\Omega }I_{1}\;dx.$$ (18)

Therefore, we have

$${ℒ}_{1}(t)+{ℒ}_{2}(t)\leq -\mu \frac{{\left(d_{1}+d_{2}\right)}^{2}}{2d_{2}}\int_{\Omega }{\left(S_{1}-\frac{\Lambda }{\mu }\right)}^{2}\;dx-\left(\nu_{2}+\mu +\sigma \right)\int_{\Omega }I_{1}^{2}\;dx\;\;+\left(\beta \varphi^{\prime }\left(0\right)-\nu_{1}\nu_{2}\left(d_{1}+d_{2}\right)-2\mu -\sigma \right)\int_{\Omega }S_{1}I_{1}\;dx\;\;+\frac{\Lambda }{\mu }\left(\beta \varphi^{\prime }\left(0\right)\left(\frac{{\left(d_{1}+d_{2}\right)}^{2}}{2d_{1}d_{2}}+\frac{2\mu }{\mu +\sigma }\right)-\mu \right)\int_{\Omega }I_{1}\;dx\;\leq \varsigma {𝖫}_{1}\left(t\right),$$ (19)

where ς is a non-negative constant defined as:


$$\varsigma =\max\backslash \{\beta \varphi^{\prime }\left(0\right)-\nu_{1}\nu_{2}\left(d_{1}+d_{2}\right)-2\mu -\sigma ,\;\;\frac{\mu +\sigma }{2\mu }\left(\beta \varphi^{\prime }\left(0\right)\left(\frac{{\left(d_{1}+d_{2}\right)}^{2}}{2d_{1}d_{2}}+\frac{2\mu }{\mu +\sigma }\right)-\mu \right)\backslash \}.$$

By Lemma 1 and Definition 1,we can conclude that:

$$\left\|L_{1}\left(t\right)\right\|\leq \left\|L_{1}\left(0\right)\right\|+\varsigma \int_{0}^{t}\left\|L_{1}\left(s\right)\right\|\;ds\leq \delta +\varsigma \int_{0}^{t}\left\|L_{1}\left(s\right)\right\|\;ds,$$ (20)

which implies

$$\left\|L_{1}\left(t\right)\right\|\leq F\left(t\right)=\delta e^{\varsigma t}.$$ (21)

Hence, the settling time can be expressed as:

$${t_{1}}^{*}=\frac{1}{\varsigma }\ln\left(\frac{\varepsilon }{\delta }\right).$$ (22)

This completes the proof.

**Theorem 2.** The EP $E_{1}^{*}$ of system (1) is FTS if :

$$S_{1}\geq S_{1}^{*},\;I_{1}\geq I_{1}^{*},$$ (23)

and

$$\max\left\{\beta C\left(\frac{I_{1}^{*}}{S_{1}^{*}}+2\right)-\mu ,\beta C\left(\frac{S_{1}^{*}}{I_{1}^{*}}+2\right)-\left(\mu +\sigma \right)\right\}>0.$$ (24)

The settling time for FTS is defined as:

$$t_{2}^{*}=\frac{1}{2\max\left\{\beta C\left(\frac{I_{1}^{*}}{S_{1}^{*}}+2\right)-\mu ,\beta C\left(\frac{S_{1}^{*}}{I_{1}^{*}}+2\right)-\left(\mu +\sigma \right)\right\}}\ln\left(\frac{\varepsilon }{\delta }\right).$$ (25)

*C* is as defined in Lemma 3.

**Proof 3.** Consider the following positive definite function:

$$L_{2}\left(t\right)=t-1-\ln\left(t\right),\;\forall t>0.$$ (26)

Let be a LF defined by:

$$L_{2}\left(t\right)=\int_{\Omega }S_{1}^{*}L_{2}\left(\frac{S_{1}}{S_{1}^{*}}\right)+I_{1}^{*}L_{2}\left(\frac{I_{1}}{I_{1}^{*}}\right)\;dx.$$ (27)

Using Lemmas 2 and 3, we obtain:


$$\frac{\partial L_{2}\left(t\right)}{\partial t}=\int_{\Omega }S_{1}^{*}\frac{\partial }{\partial t}L_{1}\left(\frac{S_{1}}{S_{1}^{*}}\right)\;dx+\int_{\Omega }I_{1}^{*}\frac{\partial }{\partial t}L_{2}\left(\frac{I_{1}}{I_{1}^{*}}\right)\;dx$$

$$=\int_{\Omega }\left(1-\frac{S_{1}^{*}}{S_{1}}\right)\frac{\partial S_{1}\left(x,t\right)}{\partial t}\;dx+\int_{\Omega }\left(1-\frac{I_{1}^{*}}{I_{1}}\right)\frac{\partial I_{1}\left(x,t\right)}{\partial t}\;dx$$

$$=\int_{\Omega }\left(1-\frac{S_{1}^{*}}{S_{1}}\right)\left(\frac{\partial {S\left(x,t\right)}_{1}}{\partial t}-\frac{\partial S_{1}^{*}}{\partial t}\right)\;dx+\int_{\Omega }\left(1-\frac{I_{1}^{*}}{I_{1}}\right)\left(\frac{\partial I_{1}\left(x,t\right)}{\partial t}-\frac{\partial I_{1}^{*}}{\partial t}\right)\;dx$$

$$=\int_{\Omega }\left(1-\frac{S_{1}^{*}}{S_{1}}\right)\left[d_{1}\Delta \left(S_{1}-S_{1}^{*}\right)-\beta \left(\frac{S_{1}\varphi \left(I_{1}\right)}{S_{1}+I_{1}}-\frac{S_{1}^{*}\varphi \left(I_{1}^{*}\right)}{S_{1}^{*}+I_{1}^{*}}\right)-\mu \left(S_{1}-S_{1}^{*}\right)\right]\;dx\;\;+\int_{\Omega }\left(1-\frac{I_{1}^{*}}{I_{1}}\right)\left[d_{2}\Delta \left(I_{1}-I_{1}^{*}\right)+\beta \left(\frac{S_{1}\varphi \left(I_{1}\right)}{S_{1}+I_{1}}-\frac{S_{1}^{*}\varphi \left(I_{1}^{*}\right)}{S_{1}^{*}+I_{1}^{*}}\right)-\left(\mu +\sigma \right)\left(I_{1}-I_{1}^{*}\right)\right]\;dx$$

$$\leq -\;d_{1}S_{1}^{*}\int_{\Omega }\frac{{\left|\nabla S_{1}\right|}^{2}}{S_{1}^{2}}\;dx-d_{2}I_{1}^{*}\int_{\Omega }\frac{{\left|\nabla I_{1}\right|}^{2}}{I_{1}^{2}}\;dx+\mu S_{1}^{*}\int_{\Omega }\left(1-\frac{S_{1}^{*}}{S_{1}}\right)\left(1-\frac{S_{1}}{S_{1}^{*}}\right)\;dx\;\;+\left(\mu +\sigma \right)I_{1}^{*}\int_{\Omega }\left(1-\frac{I_{1}^{*}}{I_{1}}\right)\left(1-\frac{I_{1}}{I_{1}^{*}}\right)\;dx\;\;\times \;\beta \int_{\Omega }\left(\left|1-\frac{S_{1}^{*}}{S_{1}}\right|+\left|1-\frac{I_{1}^{*}}{I_{1}}\right|\right)\left|\frac{S_{1}\varphi \left(I_{1}\right)}{S_{1}+I_{1}}-\frac{S_{1}^{*}\varphi \left(I_{1}^{*}\right)}{S_{1}^{*}+I_{1}^{*}}\right|\;dx$$

This, consequently, yields


$$\frac{\partial L_{2}\left(t\right)}{\partial t}\leq \mu S_{1}^{*}\int_{\Omega }\left(1-\frac{S_{1}^{*}}{S_{1}}\right)\left(1-\frac{S_{1}}{S_{1}^{*}}\right)\;dx+\left(\mu +\sigma \right)I_{1}^{*}\int_{\Omega }\left(1-\frac{I_{1}^{*}}{I_{1}}\right)\left(1-\frac{I_{1}}{I_{1}^{*}}\right)\;dx\;\;+\beta C\int_{\Omega }\left(\left|1-\frac{S_{1}^{*}}{S_{1}}\right|+\left|1-\frac{I_{1}^{*}}{I_{1}}\right|\right)\left(\left|S_{1}^{*}\right|\left|1-\frac{S_{1}}{S_{1}^{*}}\right|+\left|I_{1}^{*}\right|\left|1-\frac{I_{1}}{I_{1}^{*}}\right|\right)\;dx$$

$$=\left(\beta C-\mu \right)S_{1}^{*}\int_{\Omega }\left[L(\frac{S_{1}}{S_{1}^{*}})+L(\frac{S_{1}^{*}}{S_{1}})\right]\;dx\;\;+\left(\beta C-\mu -\sigma \right)I_{1}^{*}\int_{\Omega }\left[L(\frac{I_{1}}{I_{1}^{*}})+L(\frac{I_{1}^{*}}{I_{1}})\right]\;dx\;\;+\beta CI_{1}^{*}\int_{\Omega }\left|L(\frac{S_{1}^{*}}{S_{1}})+L(\frac{I_{1}}{I_{1}^{*}})-L(\frac{I_{1}S_{1}^{*}}{S_{1}I_{1}^{*}})\right|\;dx\;\;+\beta CS_{1}^{*}\int_{\Omega }\left|L(\frac{S_{1}}{S_{1}^{*}})+L(\frac{I_{1}^{*}}{I_{1}})-L(\frac{S_{1}I_{1}^{*}}{I_{1}S_{1}^{*}})\right|\;dx$$

$$\leq \left[\beta C\left(\frac{I_{1}^{*}}{S_{1}^{*}}+2\right)-\mu \right]\int_{\Omega }\left[S_{1}^{*}L(\frac{S_{1}}{S_{1}^{*}})+S_{1}^{*}L(\frac{S_{1}^{*}}{S_{1}})\right]\;dx\;\;+\left[\beta C\left(\frac{S_{1}^{*}}{I_{1}^{*}}+2\right)-\left(\mu +\sigma \right)\right]\int_{\Omega }\left[I_{1}^{*}L(\frac{I_{1}}{I_{1}^{*}})+I_{1}^{*}L(\frac{I_{1}^{*}}{I_{1}})\right]\;dx$$

$$\leq 2\left[\beta C\left(\frac{I_{1}^{*}}{S_{1}^{*}}+2\right)-\mu \right]\int_{\Omega }S_{1}^{*}L(\frac{S_{1}}{S_{1}^{*}})dx+2\left[\beta C\left(\frac{S_{1}^{*}}{I_{1}^{*}}+2\right)-\left(\mu +\sigma \right)\right]\int_{\Omega }(\frac{I_{1}^{*}LI_{1}}{I_{1}^{*}})dx$$

$$\leq 2\max\left\{\beta C\left(\frac{I_{1}^{*}}{S_{1}^{*}}+2\right)-\mu ,\beta C\left(\frac{S_{1}^{*}}{I_{1}^{*}}+2\right)-\left(\mu +\sigma \right)\right\}L\left(t\right).$$ (28)

Hence, we conclude:

$$\left\|L_{2}\left(t\right)\right\|\leq \delta +2\max\left\{\beta C\left(\frac{I_{1}^{*}}{S_{1}^{*}}+2\right)-\mu ,\beta C\left(\frac{S_{1}^{*}}{I_{1}^{*}}+2\right)-\left(\mu +\sigma \right)\right\}\int_{0}^{t}\left\|L\left(s\right)\right\|\;ds.$$ (29)

Utilizing Lemma 1, we obtain:

$$\left\|L_{2}\left(t\right)\right\|\leq H\left(t\right)=\delta e^{2\max\left\{\beta C\left(\frac{I_{1}^{*}}{S_{1}^{*}}+2\right)-\mu ,\beta C\left(\frac{S_{1}^{*}}{I_{1}^{*}}+2\right)-\left(\mu +\sigma \right)\right\}t}\leq \varepsilon .$$ (30)

Thus, the setting time is given by:


$${t_{2}}^{*}=\frac{1}{2\max\left\{\beta C\left(\frac{I_{1}^{*}}{S_{1}^{*}}+2\right)-\mu ,\beta C\left(\frac{S_{1}^{*}}{I_{1}^{*}}+2\right)-\left(\mu +\sigma \right)\right\}}\ln\left(\frac{\varepsilon }{\delta }\right).$$

Therefore, by Definition 1, it can be concluded that system (1) achieves stability within a finite duration, provided ${t\geq t}^{*}$.

## Finite-time synchronization scheme

This section presents a synchronization framework for master-slave RDs, focusing on achieving FTSYN. The proposed scheme ensures rapid convergence of the slave system’s states to those of the master system within a finite time. By employing LFs and designing state-dependent control laws, the approach addresses synchronization discrepancies robustly, suitable for complex nonlinear systems.

We delve into the FTSYN dynamics of the master-slave systems (1) and (31), where the slave system (31) is described by:

$$\left\{ \begin{array}{cc} \frac{\partial S_{2}\left(x,t\right)}{\partial t}=d_{1}\Delta S_{2}+\Lambda -\beta \frac{S_{2}\varphi \left(I_{2}\right)}{S_{2}+I_{2}}-\mu S_{2}+C_{1}\left(x,t\right), & x\in \Omega ,t>0,\\ \frac{\partial I_{2}\left(x,t\right)}{\partial t}=d_{2}\Delta I_{2}+\beta \frac{S_{2}\varphi \left(I_{2}\right)}{S_{2}+I_{2}}-\left(\mu +\sigma \right)I_{2}+C_{2}\left(x,t\right), & x\in \Omega ,t>0,\\ \frac{\partial S_{2}}{\partial \tau }=\frac{\partial I_{2}}{\partial \tau }=0, & x\in \partial \Omega ,\\ S_{2}\left(x,0\right)=S_{2,0}\left(x\right)>0,\;I_{2}\left(x,0\right)=I_{2,0}\left(x\right)>0, & x\in \Omega . \end{array}\right.$$ (31)

The control systems ${ℭ}_{1}(x,t)$ and ${ℭ}_{2}(x,t)$ play a vital role in achieving FTSYN in the master-slave RDs. Specifically, they are designed to ensure that the states of the slave system synchronize with those of the master system within a finite time. These control functions are incorporated into the equations governing the slave system to address synchronization discrepancies.

Their key contributions include:

- By employing feedback mechanisms, the control terms ${ℭ}_{1}(x,t)$ and ${ℭ}_{2}(x,t)$ ensure that the error terms converge to zero, leading to synchronization.
- The controllers are designed based on LF and stability criteria to guarantee that synchronization is achieved within a finite period. This rapid convergence is essential for systems requiring precise and timely synchronization.
- The control strategies are adaptable to the nonlinear and spatially extended nature of RDs, addressing the complexities of these models.

The choice and formulation of ${ℭ}_{1}$ and ${ℭ}_{2}$ are critical, as highlighted in the provided equations and proofs, where specific conditions and feedback laws are derived to achieve FTSYN efficiently.

We address the synchronization discrepancies present in Eqs (1) and (31):

$$e\left(x,t\right)=(\begin{array}{c} e_{1}\\ e_{2} \end{array})=(\begin{array}{c} S_{2}-S_{1}\\ I_{2}-I_{1} \end{array}).$$ (32)

We aim to demonstrate that the discrepancy tends to zero as time approaches ${\text{t}}^{*}$. This is accomplished by substituting the expression derived from Eq (1) into the error system delineated in Eq (33):

$$\left\{ \begin{array}{cc} \frac{\partial e_{1}\left(x,t\right)}{\partial t}=d_{1}\Delta e_{1}-\beta \left(\frac{S_{2}\varphi \left(I_{2}\right)}{S_{2}+I_{2}}-\frac{S_{1}\varphi \left(I_{1}\right)}{S_{1}+I_{1}}\right)-\mu e_{1}+C_{1}\left(x,t\right), & x\in \Omega ,t>0,\\ \frac{\partial e_{2}\left(x,t\right)}{\partial t}=d_{2}\Delta e_{2}+\beta \left(\frac{S_{2}\varphi \left(I_{2}\right)}{S_{2}+I_{2}}-\frac{S_{1}\varphi \left(I_{1}\right)}{S_{1}+I_{1}}\right)-\left(\mu +\sigma \right)e_{2}+C_{2}\left(x,t\right), & x\in \Omega ,t>0,\\ \frac{\partial e_{1}}{\partial \tau }=\frac{\partial e_{2}}{\partial \tau }=0, & x\in \partial \Omega ,\\ e_{1}\left(x,0\right)=S_{2,0}\left(x\right)-S_{1,0}\left(x\right),\;e_{2}\left(x,0\right)=I_{2,0}\left(x\right)-I_{1,0}\left(x\right), & x\in \Omega . \end{array}\right.$$ (33)

**Theorem 3.** [44] $\left(e_{1}^{*},e_{2}^{*}\right)$ *is a FTS EP of the nonlinear system (31) if there exists a positive definite LF $L_{3}:\left[0,+\infty \right)\times \Omega \to {\mathbb{R}}_{+},$ three class ℳ functions $\zeta_{1},\zeta_{2},\kappa ,$ and δ>0 such that:*

1.
$\zeta_{1}\left\|e\left(t\right)\right\|\leq L\left(t,e\left(t\right)\right)\leq \zeta_{2}\left\|e\left(t\right)\right\|.$

2.
$\frac{\partial L_{3}\left(t\right)}{\partial t}<-\kappa L_{3}\left(t,e\left(t\right)\right).$

3.
$\int_{0}^{\varepsilon }\frac{de}{\kappa \left(e\right)}<+\infty ,\;(\forall \varepsilon :0<\varepsilon \leq \delta ).$

**Definition 2.** [45,46] *The systems (1) and (31) are said to be FTSYN if there exists a settling time ${\text{t}}^{*}>0$ such that:*

$$\lim_{t\to t^{*}}\left(\|e_{1}\left(t\right)\|+\|e_{2}\left(t\right)\|\right)=0,$$ (34)

and for all $t\geq t^{*}$,

$$\|e_{1}\left(t\right)\|+\|e_{2}\left(t\right)\|\equiv 0.$$ (35)

**Theorem 4.** *The systems described by Eqs (1) and (31) achieve FTSYN by implementing the following linear feedback controller:*

$$\left\{ \begin{array}{c} {ℭ}_{1}(x,t)=-C\beta \left({𝚎}_{1}+{𝚎}_{2}\right),\\ {ℭ}_{2}(x,t)=-C\beta \left({𝚎}_{1}+{𝚎}_{2}\right), \end{array}\right.$$ (36)

where *C* is as defined in Lemma 3. The settling time of FTSYN is given by:

$$t_{3}^{*}=\frac{1}{2\min\left\{d_{1}\nu_{1}+\mu ,d_{2}\nu_{2}+\mu \right\}}\ln\left(\frac{\varepsilon }{\delta }\right).$$ (37)

**Proof 4.** We have chosen a LF represented by:

$$L_{3}\left(t\right)=\frac{1}{2}\int_{\Omega }e_{1}^{2}+e_{2}^{2}\;dx.$$ (38)

Using Lemmas 2–3 and Green’s formula, we can calculate $\frac{\partial L_{3}\left(t\right)}{\partial t}$, leading us to conclude:


$$\frac{\partial L_{3}\left(t\right)}{\partial t}=\int_{\Omega }e_{1}\frac{\partial e_{1}\left(x,t\right)}{\partial t}\;dx+\int_{\Omega }e_{2}\frac{\partial e_{2}\left(x,t\right)}{\partial t}\;dx$$

$$=\int_{\Omega }e_{1}\left[d_{1}\Delta e_{1}-\beta \left(\frac{S_{2}\varphi \left(I_{2}\right)}{S_{2}+I_{2}}-\frac{S_{1}\varphi \left(I_{1}\right)}{S_{1}+I_{1}}\right)-\mu e_{1}-C\beta \left(e_{1}+e_{2}\right)\right]\;dx\;\;+\int_{\Omega }e_{2}\left[d_{2}\Delta e_{2}+\beta \left(\frac{S_{2}\varphi \left(I_{2}\right)}{S_{2}+I_{2}}-\beta \frac{S_{1}\varphi \left(I_{1}\right)}{S_{1}+I_{1}}\right)-\left(\mu +\sigma \right)e_{2}-C\beta \left(e_{1}+e_{2}\right)\right]\;dx$$

$$\leq \int_{\Omega }e_{1}\left[d_{1}\Delta e_{1}-\beta \left|\frac{S_{2}\varphi \left(I_{2}\right)}{S_{2}+I_{2}}-\frac{S_{1}\varphi \left(I_{1}\right)}{S_{1}+I_{1}}\right|-\mu e_{1}-C\beta \left(e_{1}+e_{2}\right)\right]\;dx\;\;+\int_{\Omega }e_{2}\left[d_{2}\Delta e_{2}+\beta \left|\frac{S_{2}\varphi \left(I_{2}\right)}{S_{2}+I_{2}}-\beta \frac{S_{1}\varphi \left(I_{1}\right)}{S_{1}+I_{1}}\right|-\left(\mu +\sigma \right)e_{2}-C\beta \left(e_{1}+e_{2}\right)\right]\;dx$$

$$\leq \int_{\Omega }e_{1}\left[d_{1}\Delta e_{1}+C\beta \left(e_{1}+e_{2}\right)-\mu e_{1}-C\beta \left(e_{1}+e_{2}\right)\right]\;dx\;\;+\int_{\Omega }e_{2}\left[d_{2}\Delta e_{2}+C\beta \left(e_{1}+e_{2}\right)-\left(\mu +\sigma \right)e_{2}-C\beta \left(e_{1}+e_{2}\right)\right]\;dx$$

$$=\int_{\Omega }e_{1}\left[d_{1}\Delta e_{1}-\mu e_{1}\right]\;dx+\frac{1}{2}\int_{\Omega }e_{2}\left[d_{2}\Delta e_{2}-\left(\mu +\sigma \right)e_{2}\right]\;dx$$

$$\leq -d_{1}\int_{\Omega }{\left|\nabla e_{1}\right|}^{2}\;dx-d_{2}\int_{\Omega }{\left|\nabla e_{2}\right|}^{2}\;dx-\mu \int_{\Omega }e_{1}^{2}\;dx-\mu \int_{\Omega }e_{2}^{2}\;dx$$

$$\leq -2\min\left\{d_{1}\nu_{1}+\mu ,d_{2}\nu_{2}+\mu \right\}L_{3}\left(t\right).$$ (39)

By defining $\kappa \left(e\right)=2\min\left\{d_{1}\nu_{1}+\mu ,d_{2}\nu_{2}+\mu \right\}$, we obtain :

$$\int_{0}^{\varepsilon }\frac{de}{\kappa \left(e\right)}=\frac{\varepsilon }{2\min\left\{d_{1}\nu_{1}+\mu ,d_{2}\nu_{2}+\mu \right\}}<+\infty ,$$ (40)

Using Theorem 1, we establish that the zero solution of the error system (33) signifies the FTS of the EP $\left(e_{1}^{*},e_{2}^{*}\right)=\left(0,0\right)$. Thus, we have:

$$L_{3}(t)\leq L_{3}(0)-2\min\left\{d_{1}\nu_{1}+\mu ,d_{2}\nu_{2}+\mu \right\}\int_{0}^{t}L_{3}(s)\;ds.$$ (41)

This implies

$$\left\|L_{3}(t)\right\|\leq \delta +2\min\left\{d_{1}\nu_{1}+\mu ,d_{2}\nu_{2}+\mu \right\}\int_{0}^{t}\left\|L_{3}(s)\right\|\;ds.$$ (42)

Applying Lemma 1, we deduce the following inequality:

$$\left\|L_{3}(t)\right\|\leq G\left(t\right)=\delta e^{2\min\left\{d_{1}\nu_{1}+\mu ,d_{2}\nu_{2}+\mu \right\}t}.$$ (43)

Finally, the synchronization time is estimated as :

$$t_{3}^{*}=\frac{1}{2\min\left\{d_{1}\nu_{1}+\mu ,d_{2}\nu_{2}+\mu \right\}}\ln\left(\frac{\varepsilon }{\delta }\right).$$ (44)

Consequently, according to the criteria specified in Definition 2, the systems described by (Eqs 1) and (31) achieve synchronization within a finite time $t_{3}^{*}$.

## Numerical simulations

To validate the theoretical findings, this section presents numerical simulations of the proposed stability and synchronization methods. Examples illustrating the dynamic behavior of RDs are provided, with parameters tailored to demonstrate finite-time convergence. The results are visualized through spatiotemporal plots and LF trajectories, showcasing the practical applicability and accuracy of the developed methodologies.

**Example 1.** *In the specified domain x∈[0,10] and t∈[0,5], the parameter values are set as follows:*

$$(d_{1},d_{2},\Lambda ,\beta ,\mu ,\sigma ,\nu_{1},\nu_{2},N)=\left(1.5,1.5,5,0.75,2,3.891,0.01,0.01,100\right)$$ (45)

*The initial conditions are defined as :*

$$S_{1,0}\left(x\right)=1,\;I_{1,0}\left(x\right)=2.$$ (46)

*The function $\varphi (I_{1})$ is given by:*

$$\varphi (I_{1})=\frac{I_{1}}{1+I_{1}},$$ (47)

*with satisfies the Lipschitz condition:*

$$\left|\varphi (I_{1})-\varphi (I_{2})\right|\leq \left|I_{1}-I_{2}\right|,$$ (48)

*and remains uniformly bounded :*

$$\left|\varphi \left(I_{1}\right)\right|=\left|\frac{I_{1}}{1+I_{1}}\right|\leq 1,$$ (49)

*From the setup, the parameters δ and ε are determined as:*

δ=9.712009336275674,ε=10.395009336275674. (50)

*The stability condition of Theorem 1 is satisfied:*

$$\max\left\{\Xi^{*}\right\}=0.013625>0,$$ (51)

*where*


$$\Xi^{*}=\beta \varphi^{\prime }\left(0\right)-\nu_{1}\nu_{2}\left(d_{1}+d_{2}\right)-2\mu -\sigma ,\frac{\mu +\sigma }{2\mu }\left(\beta \varphi^{\prime }\left(0\right)\left(\frac{{\left(d_{1}+d_{2}\right)}^{2}}{2d_{1}d_{2}}+\frac{2\mu }{\mu +\sigma }\right)-\mu \right).$$

*The settling time is calculated as:*

$${t_{1}}^{*}=\frac{1}{\max\left\{\Xi^{*}\right\}}\ln\left(\frac{\varepsilon }{\delta }\right)=4.988082482944463s.$$ (52)

**Fig 2:**
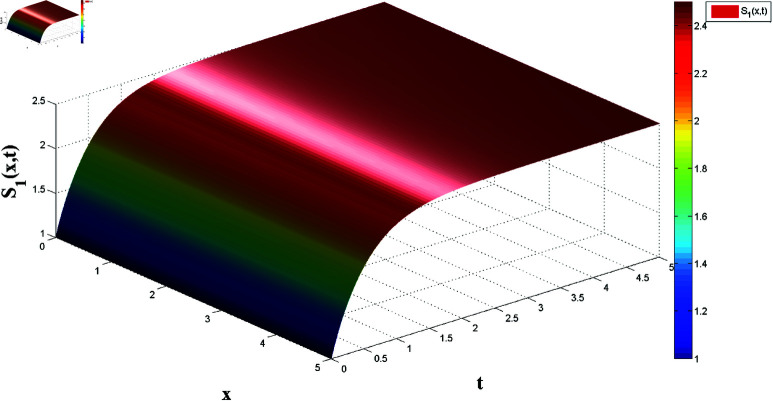
Spatial dynamics of solution $S_{1}(x,t)$.

**Fig 3:**
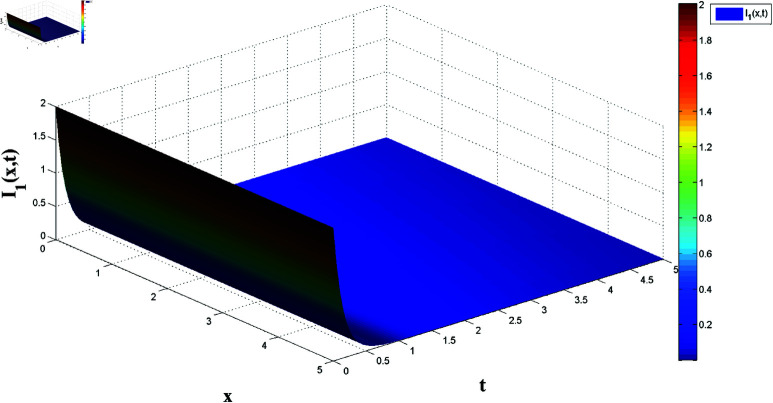
Temporal dynamics of solutions $I_{1}(x,t)$.

*Figs 2 and 3 present, respectively, the solutions $S_{1}(x,t)$ and $I_{1}(x,t)$ over space and time, demonstrating the dynamics under homogeneous Neumann boundary conditions. The EP $\left(S_{1}^{*},I_{1}^{*})=(2.5,0\right)$ is determined based on Theorem 1, confirming the system’s FTS.*

**Fig 4:**
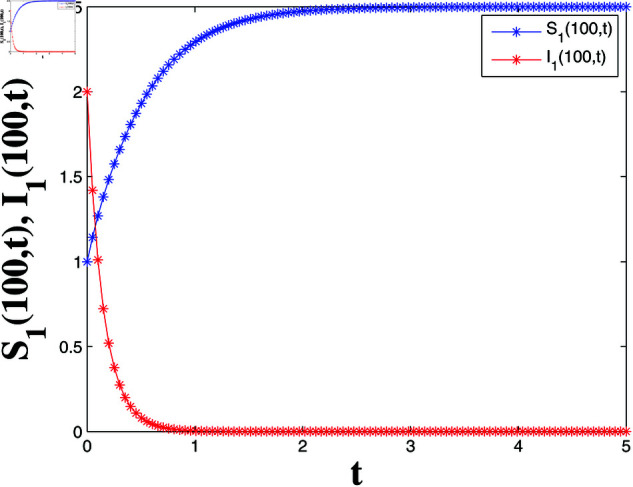
State trajectories of solutions $S_{1}\left(100,t\right)$ and $I_{1}\left(100,t\right)$.

**Fig 5:**
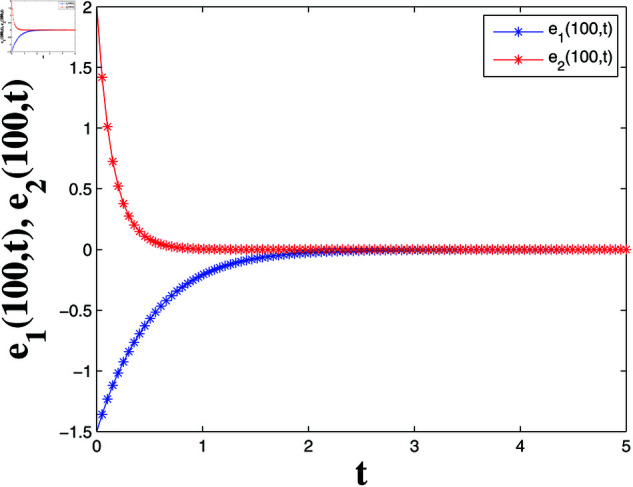
Error dynamics of solutions $S_{1}\left(100,t\right)$ and $I_{1}\left(100,t\right)$.

**Fig 6:**
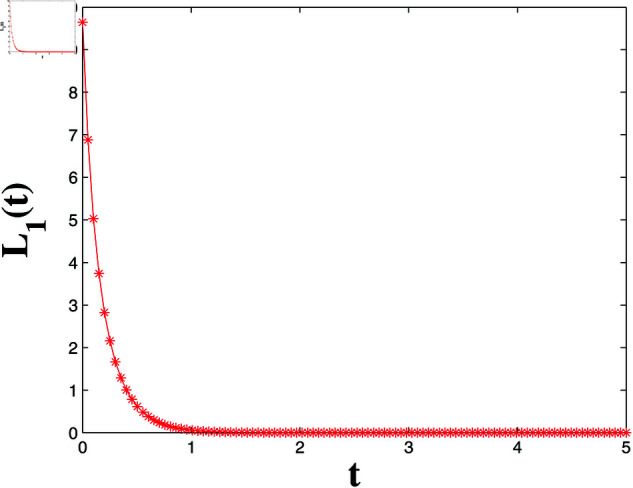
Estimation of the LF $L_{1}(t)$.

**Fig 7:**
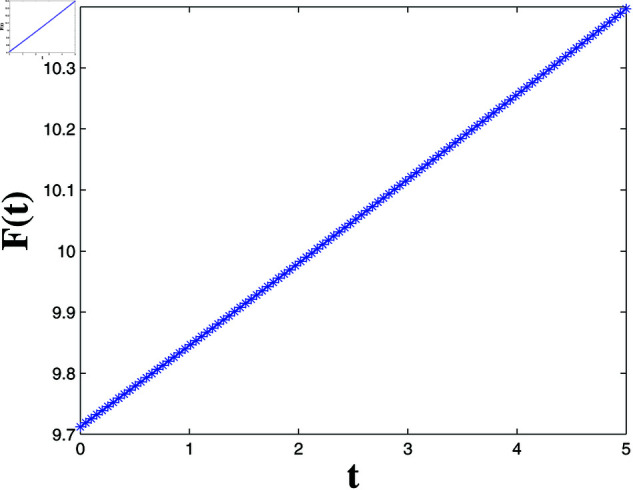
Estimation of the LF F(t).

*Figs 4, 5, 6, and 7 depict numerical validation, showing that errors and LF converge to zero as t approaches $t_{1}^{*}=4.988082482944463s$.*

**Example 2.** *Consider the intervals x∈[0,10] and t∈[0,10]. The parameters for this example are chosen as*

$$\left(d_{1},d_{2},\Lambda ,\beta ,\mu ,\sigma ,C,N\right)=\left(0.1,0.1,1.5,0.1092,3,0.149775,2,100\right).$$ (53)

*The initial conditions are given by:*

$$S_{1,0}\left(x\right)=3,\;I_{1,0}\left(x\right)=4.5.$$ (54)

*The function $\varphi (I_{1})$ is redefined for this scenario as:*

$$\varphi \left(I_{1}\right)=\frac{I_{1}}{I_{1}+S_{1}}$$ (55)

*This function satisfies the Lipschitz condition:*

$$\left|\varphi \left(I_{1}\right)-\varphi \left(I_{2}\right)\right|=\left|\frac{I_{1}}{I_{1}+S_{1}}-\frac{I_{2}}{I_{2}+S_{1}}\right|\leq \left|I_{1}-I_{2}\right|,$$ (56)

*and is uniformly bounded as:*

$$\left|\varphi \left(I_{1}\right)\right|=\left|\frac{I_{1}}{I_{1}+S_{1}}\right|\leq 1,$$ (57)

*For this configuration, the computed values of δ and ε are:*

δ=5.895588996582122,ε=6.828588996582122, (58)

*The stability condition outlined in Theorem 2 is verified with*

$$S_{1}\geq 0.5197,\;I_{1}\geq 0.0436,\;$$ (59)

*and*


$$\max\left\{\beta C\left(\frac{I_{1}^{*}}{S_{1}^{*}}+2\right)-\mu ,\beta C\left(\frac{S_{1}^{*}}{I_{1}^{*}}+2\right)-\left(\mu +\sigma \right)\right\}=0.007352292360051.$$

*The settling time for FTS is computed as:*

$$t_{2}^{*}=\frac{1}{2\max\left\{\beta C\left(\frac{I_{1}^{*}}{S_{1}^{*}}+2\right)-\mu ,\beta C\left(\frac{S_{1}^{*}}{I_{1}^{*}}+2\right)-\left(\mu +\sigma \right)\right\}}\ln\left(\frac{\varepsilon }{\delta }\right)$$ (60)

=9.991007726890397s. (61)

**Fig 8:**
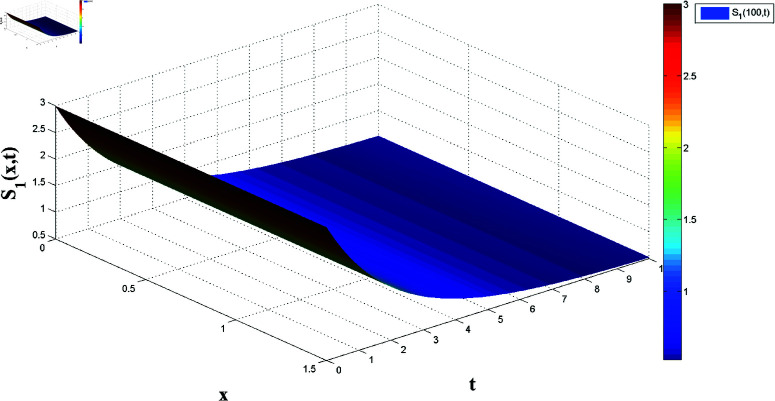
Spatiotemporal dynamics of susceptible populations.

**Fig 9:**
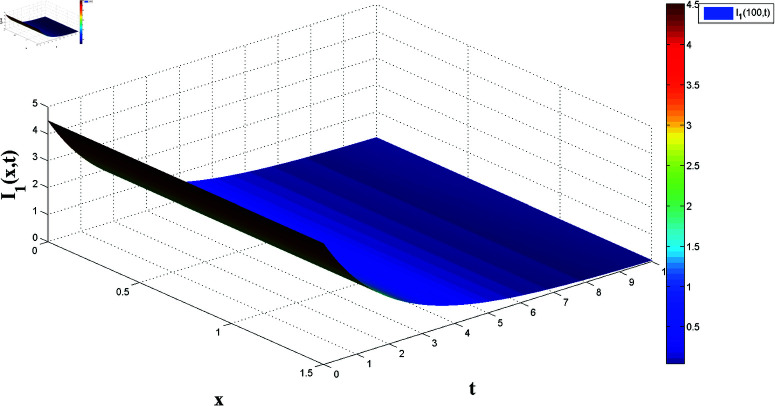
Spatiotemporal dynamics of infected populations.

**Fig 10:**
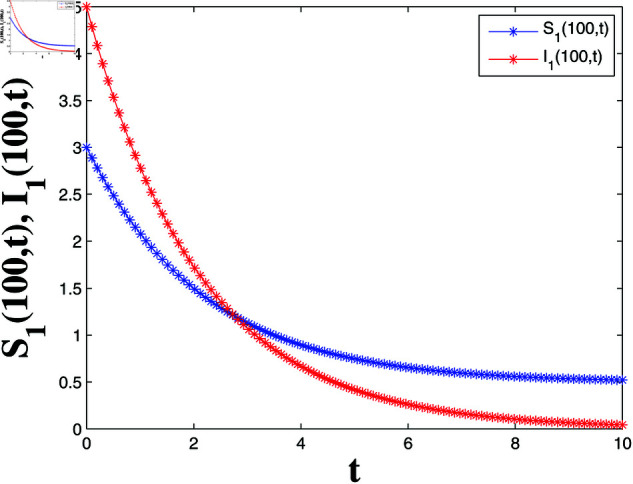
State trajectories in relation of Example 2.

**Fig 11:**
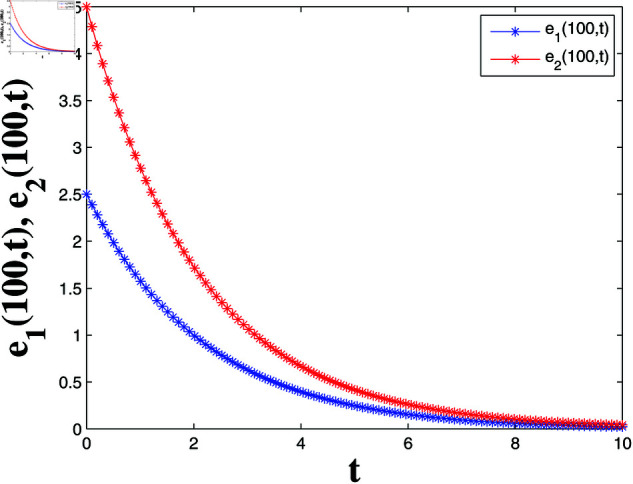
Error evolution in relation of Example 2.

*Figs 8, 9, 10, and 11 illustrate the spatiotemporal dynamics of the solutions $S_{1}$ and $I_{1}$, highlighting the system’s behavior under the homogeneous Neumann boundary conditions. An EP $(S_{1}^{*},I_{1}^{*})=\left(0.521389399120644,0.041859464884153\right)$ is identified, demonstrating the system’s FTS as per Theorem 2.*

**Fig 12:**
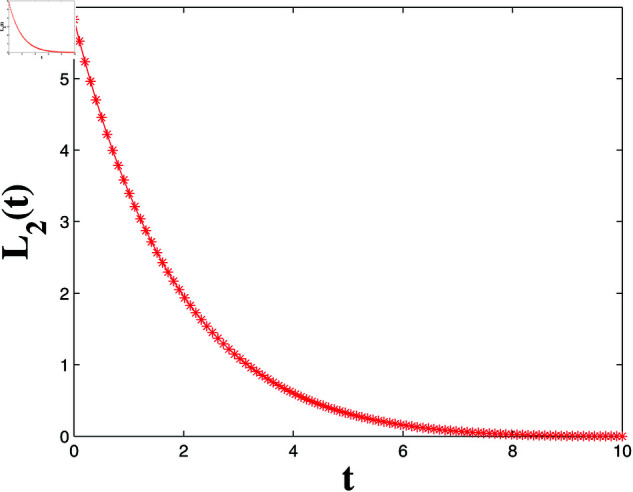
LF estimation in relation to Example 2.

**Fig 13:**
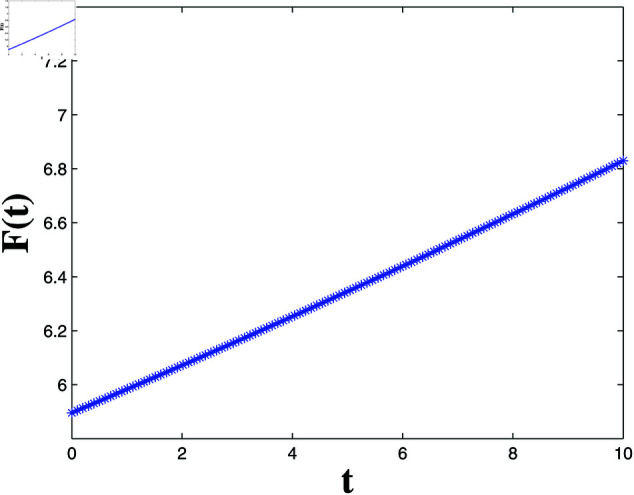
F(t) in relation to Example 2.

*Numerical simulations verify the theoretical findings, showing that the LF $L_{2}(t)$ converges to zero as time approaches $t_{2}^{*}=9.991007726890397\;\text{s}$ in Figs 12 and 13. The convergence of the error terms further corroborates the system’s FTS.*

**Example 3.** *Consider the spatial domain x∈[0,6], and temporal domain t∈[0,1]. The parameters are selected as follows:*

$$\left(d_{1},d_{2},\Lambda ,\beta ,\mu ,\sigma ,N\right)=\left(0.1,0.1,6,0.2,3.16,0.1,100\right).$$ (62)

*The initial conditions are defined as:*

$$S_{1,0}\left(x\right)=0.025-0.0025\cos\left(3x\right),\;I_{1,0}\left(x\right)=0.02-0.002\sin\left(4x\right),$$ (63)

*and*

$$S_{2,0}\left(x\right)=0.05-0.0075\sin\left(3x\right),\;I_{2,0}\left(x\right)=0.025-0.0037\cos\left(4x\right).$$ (64)

*The infection rate function $\varphi (I_{1})$ is modified to account for spatial variability:*

$$\varphi \left(I_{1}\right)=I_{1},$$ (65)

*This function satisfies the Lipschitz condition and boundedness. Then, The computed stability parameters are:*

C=1.0220,δ=0.0037,ε=2.0012. (66)

*Using Theorem 4, the stability condition is verified:*

$$\left\{ \begin{array}{c} C_{1}\left(x,t\right)=-0.2044\left(e_{1}+e_{2}\right),\\ C_{2}\left(x,t\right)=-0.2044\left(e_{1}+e_{2}\right). \end{array}\right.$$ (67)

*The settling time for FTSYN is calculated as:*

$$t_{3}^{*}=\frac{1}{2\min\left\{d_{1}\nu_{1}+\mu ,d_{2}\nu_{2}+\mu \right\}}\ln\left(\frac{\varepsilon }{\delta }\right)=0.9973s.$$ (68)

*Numerical simulations were conducted to validate the FTS and FTSYN of the proposed RDs, with results presented in the subsequent figures. These visualizations provide insights into the system dynamics and synchronization behavior under specified initial conditions and parameters.*

**Fig 14:**
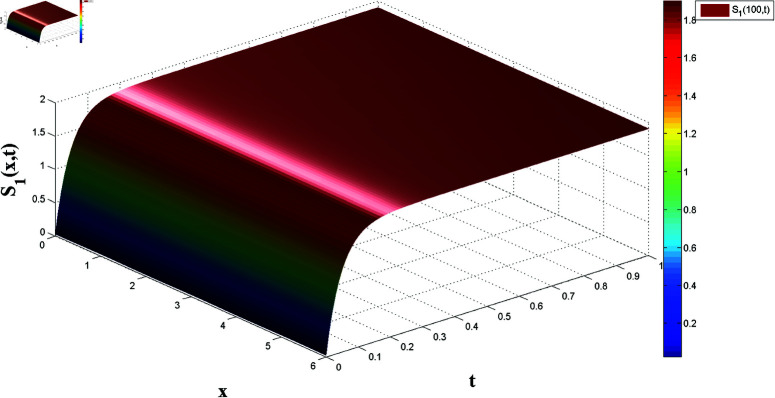
Master system dynamics: Evolution of $S_{1}(x,t)$.

**Fig 15:**
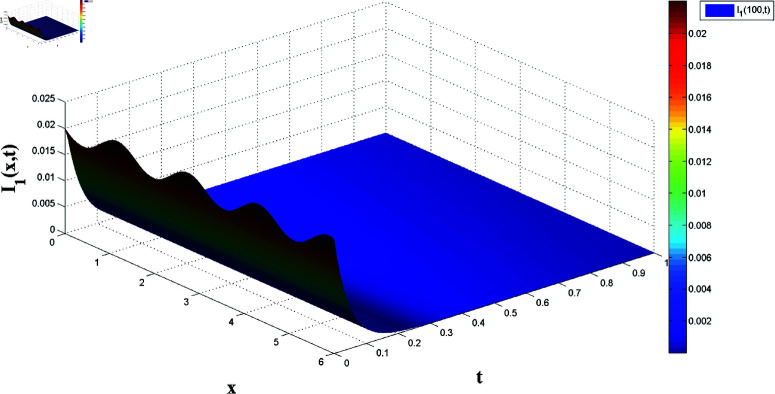
Master system dynamics: Evolution of $I_{1}(x,t)$.

**Fig 16:**
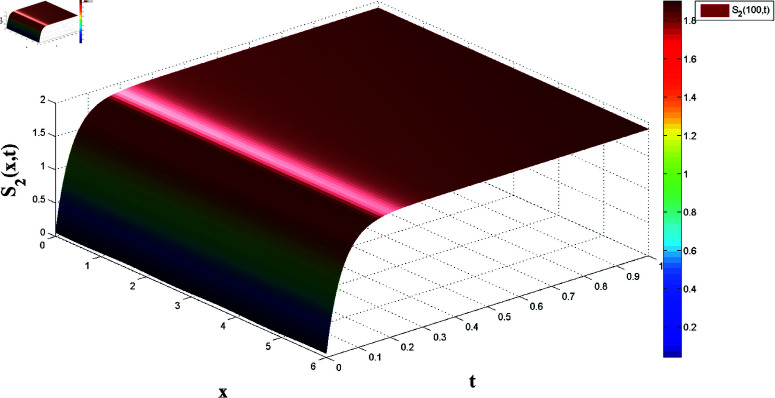
Slave system dynamics: Evolution of $S_{2}(x,t)$.

**Fig 17:**
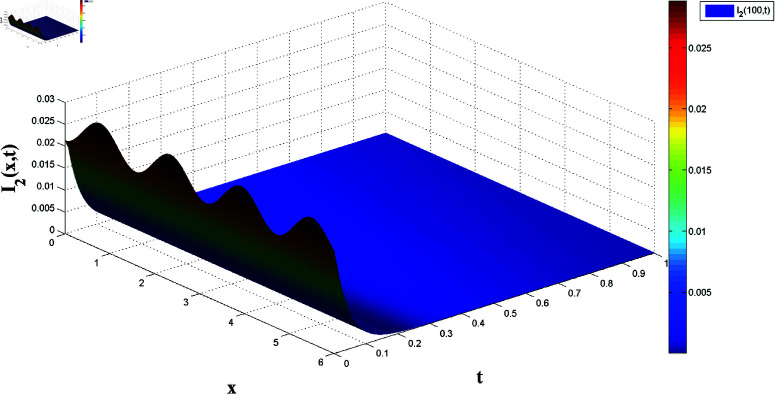
Slave system dynamics: Evolution of $I_{2}(x,t)$.

**Fig 18:**
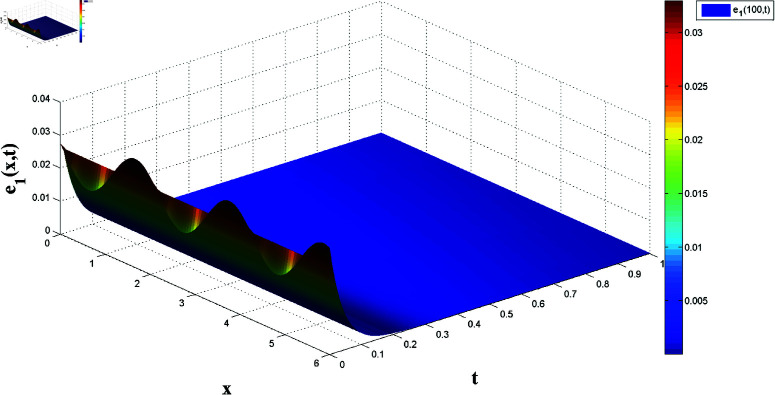
Synchronization error $e_{1}(x,t)$ convergence for master-slave systems.

**Fig 19:**
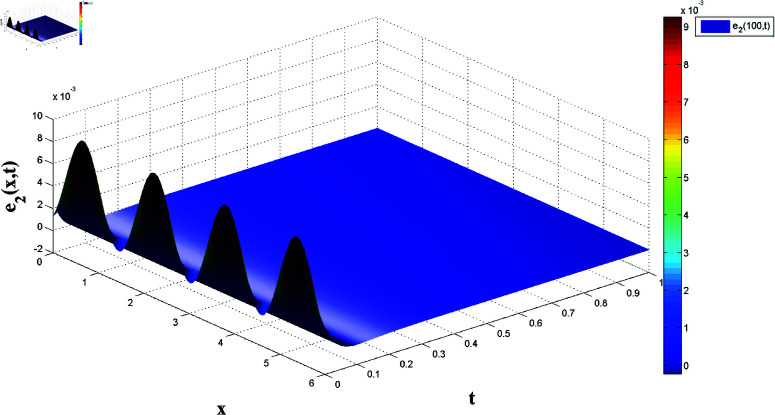
Synchronization error $e_{2}(x,t)$ convergence for master-slave systems.

**Fig 20:**
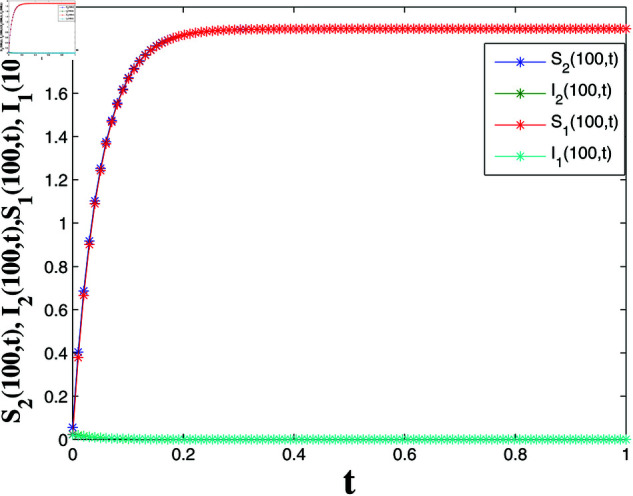
State trajectories at x=100.

**Fig 21:**
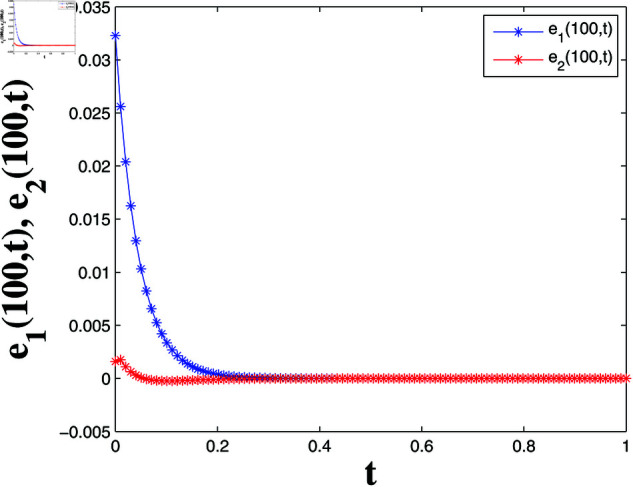
Synchronization errors at x=100.

**Fig 22:**
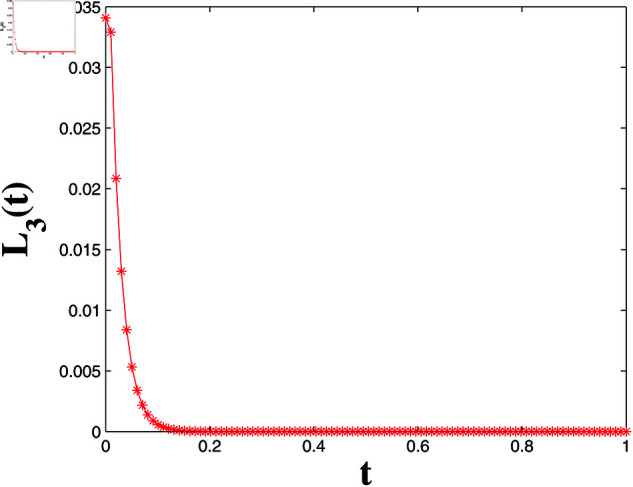
LF $L_{3}(t)$ convergence for FTSYN.

- *Figs 14 and 15 illustrate the spatiotemporal evolution of the master system’s state variables, $S_{1}$ and $I_{1}$, over x∈[0,6] and t∈[0,1], demonstrating stabilization towards the equilibrium point as predicted by Theorem 4, confirming FTS.*

- *Figs 16 and 17 show the slave system’s state variables, $S_{2}$ and $I_{2}$, synchronizing with the master system under control laws, supporting the FTSYN claim from Theorem 4.*

- *Figs 18 and 19 display synchronization errors $e_{1}$ and $e_{2}$ converging to zero as $t\to t^{*}$, verifying the control scheme’s effectiveness in achieving FTSYN.*

- *Figs 20 and 21 offer a one-dimensional perspective, plotting state trajectories and synchronization errors at *x* = 100, where rapid error decay validates FTSYN at a fixed spatial location.*

- *Fig 22 depicts the LF $L_{3}(t)$, showing monotonic decrease and convergence to zero within $t_{3}^{*}=0.9973$ s, confirming synchronization error stability and satisfying Theorem 4.*

*These results collectively confirm the theoretical findings of Example 3, demonstrating the robustness and practical applicability of the proposed methodology in SIS RDs.*

## Conclusion and future work

This study has comprehensively analyzed FTS and FTSYN in integer-order epidemic RDs, addressing significant gaps in the existing literature. By integrating LF, Gronwall’s inequality, and linear control strategies, we derived sufficient conditions for the FTS of EPs and FTSYN of master-slave systems. Numerical simulations demonstrated the effectiveness of the proposed methodologies, providing valuable insights into the dynamic behavior and control of epidemic models. The results underline the critical role of diffusion rates, interaction frequencies, and control parameters in achieving rapid stabilization and synchronization within finite time. Practical examples and MATLAB simulations validate the theoretical findings and highlight the real-world applicability of the proposed framework in modeling and managing infectious disease transmission.

Despite the advancements made, several areas remain open for further exploration:

- Extending the current framework to fractional-order RDs to account for memory and hereditary effects in dynamic processes.
- Investigating more complex control strategies, including nonlinear and time-varying approaches, to enhance system robustness against uncertainties and external perturbations.
- Applying the proposed methods to larger, more complex networks, including multi-patch epidemic models and agent-based systems.
- Incorporating stochastic elements into the models to capture the randomness and uncertainty inherent in real-world epidemic scenarios.
- Developing hardware and software implementations of the control strategies to facilitate their deployment in real-time monitoring and control systems.

This work lays a robust foundation for future research into finite-time dynamics and synchronization, with potential applications in epidemiology, ecology, and engineering.
